# Data on IL-17 production induced by plant lectins

**DOI:** 10.1016/j.dib.2016.04.053

**Published:** 2016-04-27

**Authors:** Thiago Aparecido da Silva, Fabrício Freitas Fernandes, Maria Cristina Roque-Barreira

**Affiliations:** Departamento de Biologia Celular e Molecular e Bioagentes Patogênicos, Faculdade de Medicina de Ribeirão Preto, Universidade de São Paulo, Brazil

**Keywords:** IL-17, Plant lectins, ArtinM, Carbohydrate recognition, Immunomodulation

## Abstract

We reported in article da Silva et al. (2016) [2] that ArtinM induces the IL-17 production through interaction with CD4^+^ T cells and stimulation of IL-23 and IL-1. Besides ArtinM, other plant lectins (PLs) induce IL-17 production by murine spleen cells. The IL-17 production induced by PLs was evaluated regarding the involvement of IL-23, IL-6, Th1-, and Th2-cytokines. Furthermore, the effect exerted TLR2, TLR4, and CD14 on the PLs׳ performance in the induction of IL-17 was examined. The current data were compared to the known ArtinM ability to induce Th17 immunity.

**Specification Table**

**Table:** 

Subject area	Biology
More specific subject area	Glycobiology
Type of data	Tables
How data was acquired	The data were generated from enzyme-linked immunosorbent assay (ELISA) kit for murine IL-17 measurement, which was performed according to the manufacturer׳s instructions (Ready-SET-Go Kit; eBioscience, San Diego, CA, USA). Following the reaction, microplates were read at 450 nm in a spectro- photometer (Power Wave X; BioteK Instruments). The IL-17 (pg/mL) concentration in samples was determined with reference to an absorbance curve for serial dilutions of a standard solution of murine recombinant IL-17.
Data format	Analyzed and graphed
Experimental factors	Cultures of spleen cells from C57BL/6 mice or genetically deficient C57BL/6 mice (knockout for either IFN-γ, IL-4, IL-6, IL-10, IL-22, IL-23, TLR2, TLR4, and CD14 gene) were stimulated with plant lectins (PLs) for 48 h. Cultures supernatant was used to measure the IL-17 production by ELISA.
Experimental features	IL-17 production by murine spleen cells stimulated with plant lectins with distinct specificities of sugar recognition.
Data source location	University of São Paulo, Brazil
Data acessibility	Data is within this article.

**Value of the data**

• The report of induction of Th17-immunity by lectins corroborates to the immunomodulatory potential of carbohydrate recognition, opening a new approach to confer protection against pathogens, especially against fungal diseases.
• Triggering or maintenance of IL-17 production frequently requires the involvement of cytokines and receptors on immune cells; the identification of such dependence indicates the mechanisms used by a certain lectin to modulate Th17 immunity. This feature is relevant for the choice of which cell population should be used for in vitro studies of immunomodulatory agents.
• The data demonstrate that the IL-17 production induced by lectins with distinct sugar-binding specificities instigate further studies with other lectins to analyze the induction of Th17 response, taking this report as an application guidelines.

## 1 Data

The data demonstrate that stimulation of murine spleen cells with plant lectins (PL) may induce the IL-17 production, at levels significant higher than the detected in the absence of the stimulus (Table 1, Table 2). Table 1 shows that spleen cells from IL-4 or IFN-γ KO mice under MAL-1 stimulus duplicated the IL-17 production, and the spleen cells from IL-23 KO mice reduced to 66% the levels of IL-17 in response to MAL-1 (Table 1), compared to WT mice. The spleen cells from IL-10 KO mice showed a significant increase in the IL-17 production, when induced by L-PHA, E-PHA, and ConA (Table 1), whereas Table 2 shows that spleen cells from TLR2 KO, TLR4 KO, and CD14 KO mice not affect significantly the IL-17 production induced by PL.

## 2 Experimental design, materials and methods

### 2.1 Materials

Male C57BL/6 (wild-type, WT) (provided by University of São Paulo, Ribeirão Preto, São Paulo, Brazil), IL-4 KO (JAX^®^-002518), IL-6 KO (CNRS, Orléans, France), IL-10 KO (JAX^®^-002251), IFN-γ KO (JAX^®^-002287), IL-22 KO (CNRS, Orléans, France), IL-23 KO (CNRS, Orléans, France), TLR2 KO (JAX^®^-004650), TLR4 KO (provided by Federal University of Minas Gerais – UFMG, Belo Horizonte, Brazil) and CD14 KO (JAX^®^-003726) mice at 6–8 weeks of age were used in this study. These mice were housed in the animal facility of the Molecular and Cellular Biology Department of the Faculty of Medicine of Ribeirão Preto, University of São Paulo, under optimized hygienic conditions. The Committee of Ethics in Animal Research of the College of Medicine of Ribeirão Preto at the University of São Paulo approved the animal studies, Protocol no. 082/2012.

The plant lectins were purified or purchased, as following: Jacalin were purified as previously described [1] from the saline extract of *Artocarpus heterophyllus* (jackfruit) seeds via affinity chromatography on sugar columns. The lectins *Canavalia ensiformis* (ConA), *Phaseolus vulgaris* erythroagglutinin (E-PHA), *Phaseolus vulgaris* leukoagglutinin (L-PHA), *Sambucus nigra* agglutinin (SNA), and *Maackia amurensis* leukoagglutinin (MAL-1), and *Ulex europaeus* agglutinin (UEA) were purchased from Sigma Chemical (Sigma-Aldrich, St. Louis, MO, USA).

## 3 Experimental design

The suspensions of spleen cells from mice were obtained as described in da Silva et al., [2]. These cells were obtained from all mice strains and used to measure the levels of IL-17 in the culture supernatant. The suspensions of spleen cells (2×10^6^/mL) were cultured in 96-well microplates in the presence of ConA, E-PHA, L-PHA, SNA, MAL, UEA, or Jacalin, all used in final concentrations of 2.5 µg/mL. The reference lectin, ArtinM, was added at concentration of 1.25 µg/mL, and was used phorbol myristate acetate (PMA; Sigma-Aldrich) plus ionomycin (Sigma-Aldrich) as positive control [2]. After 48 h of incubation, the spleen cells were centrifuged (300*g*, 10 min at room temperature) and the supernatants were collected to measure IL-17A levels. This quantification was determined by an enzyme-linked immunosorbent assay (ELISA) from Ready-SET-Go Kit (eBioscience) according to the manufacturer׳s instructions. After, the results were analyzed using Prism (Graph Pad Software), and the values are expressed as mean±standard error of the mean (SEM). Statistical determinations of the difference between means of groups were performed with analysis of variance (1-way) followed by Bonferroni׳s multiple comparison tests. ^*^p<0.05 were considered statistically significant.

## Conflict of interest

The authors declare that they have no competing interests.

## Figures and Tables

**Table 1:** IL-17 production induced by lectins in spleen cells obtained from C57BL/6 (WT) and knockouts for cytokines (KO) mice. The levels of IL-17 in the culture supernatants were measured by ELISA, and the values are expressed as mean ±SEM. One-way ANOVA analysis of variance followed by Bonferroni׳s post-test was used to compare WT and KO.

Lectins/Mice	WT	IL-4 KO	IL-10 KO	IFN-γ KO	IL-6 KO	IL-22 KO	IL-23 KO
Medium	4.47±0.73	6.09±0.82	6.13±1.53	5.07±0.90	6.59±1.51	7.91±1.87	9.40±4.99
ConA	1114.0±122.3	895.0±49.3	1928.0±263.6⁎	1335.0±205.7	1510.0±206.7	1230.0±149.6	945.5±187.2
E-PHA	196.8±17.5	197.8±39.2	399.7±92.1⁎	184.2±17.2	189.0±21.4	249.5±21.9	198.2±19.9
L-PHA	212.7±17.9	249.1±25.6	380.9±63.8⁎	154.8±27.9	203.9±20.9	261.1±24.2	166.3±20.3
SNA	67.7±6.2	64.6±4.5	73.8±12.4	67.9±8.2	42.2 ±4.3⁎	83.5±14.7	61.59±6.7
MAL-1	309.1±40.7	643.2±127.1⁎	503.8±148.0	620.4±142.4⁎	428.9±94.0	425.6±144.7	104.8±15.7⁎
UEA	75.3±5.0	74.4±8.3	60.4±5.7	77.8±7.4	56.8±4.2	105.4±12.2⁎	60.89±5.1
Jacalin	241.8±13.4	225.6±23.7	221.5±23.2	229.1±7.5	233.8±19.7	262.3±30.1	266.3±28.4

⁎ *p*<0.05.

**Table 2:** IL-17 production induced by lectins in spleen cells obtained from WT and KO for receptors mice. The levels of IL-17 in the culture supernatants were measured by ELISA, and the values are expressed as mean ±SEM. One-way ANOVA analysis of variance followed by Bonferroni׳s post-test was used to compare WT and KO.

Lectins/Mice	WT	TLR2 KO	TLR4 KO	CD14 KO
Medium	4.52±0.96	8.91±2.45	3.83±0.58	7.31±1.06
ConA	1137.0±151.1	1106.0±39.73	1158.0±261.7	1004.0±330.4
E-PHA	188.4±23.4	162.7±34.6	240.1±6.7	201.5±25.2
L-PHA	212.4±20.15	254.3±16.22	180.2±15.96	164.9±17.04
SNA	67.5±5.8	65.3±6.5	45.5±2.5⁎	54.9±6.3
MAL-1	359.2±51.4	994.7±68.5⁎	273.9±97.3	448.4±179.6
UEA	76.3±7.2	79.3±7.2	62.8±3.9	74.1±5.5
Jacalin	234.9±18.4	251.4±18.4	222.7±8.7	224.9±5.4

⁎ *p*<0.05.
