# Supplementary material for: Data on IL-17 production induced by plant lectins
Source: Data Brief. 2016 Apr 27;7:1584–7. doi: 10.1016/j.dib.2016.04.053 (PMC4865662; doi:10.1016/j.dib.2016.04.053)
Supplement: Supplementary file 1 — Supplementary material [file mmc1.docx]

March 7, 2016.

Dear Editor,

We would like to submit enclosed the manuscript “**Data on IL-17 production induced by plant lectins”** by Thiago Aparecido da Silva and Maria Cristina Roque-Barreira for publication as an original research article in “Data in Brief”.

We certify that all authors have seen and approved the final version of the manuscript being submitted. There is no financial interest to report and this work is not under review at any other publication.

Kind regards.

Sincerely yours,

Maria Cristina Roque Barreira, MD, PhD

Please address all correspondence to: Maria Cristina Roque Barreira, Department of Molecular and Cell Biology, School of Medicine of Ribeirão Preto, University of São Paulo, Avenida Bandeirantes 3900, 14049-900, Ribeirão Preto, São Paulo, Brazil. Phone: +55 16 3315-3062; [mcrbarre@fmrp.usp.br](mailto:mcrbarre@fmrp.usp.br)
